# Are sarcopenia and its individual components linked to all-cause mortality in heart failure? A systematic review and meta-analysis

**DOI:** 10.1007/s00392-023-02360-8

**Published:** 2023-12-12

**Authors:** Konstantinos Prokopidis, Konstantinos Katsikas Triantafyllidis, Konstantinos Spyridon Kechagias, Alexandros Mitropoulos, Rajiv Sankaranarayanan, Masoud Isanejad

**Affiliations:** 1 Department of Musculoskeletal Ageing and Science, Institute of Life Course and Medical Sciences, University of Liverpool, Liverpool, UK; 2 Department of Nutrition and Dietetics, Homerton University Hospital Foundation Trust, London, UK; 3 Department of Obstetrics and Gynaecology, The Hillingdon Hospitals NHS Foundation Trust, London, UK; 4 Lifestyle, Exercise and Nutrition Improvement (LENI) Research Group, Department of Nursing and Midwifery, Sheffield Hallam University, Sheffield, UK; 5 Liverpool Centre for Cardiovascular Science, University of Liverpool, Liverpool, UK; 6 Liverpool University Hospitals NHS Foundation Trust, Liverpool, UK; 7 National Institute for Health and Care Research, Liverpool, UK

**Keywords:** Heart failure, Sarcopenia, Handgrip strength, Appendicular lean mass, Mortality

## Abstract

**Objective:**

The objective of this systematic review and meta-analysis was to assess sarcopenia and its components as prognostic factors in patients with heart failure (HF).

**Methods:**

From inception to December 2022, a systematic literature search was carried out utilizing PubMed, Web of Science, Scopus, and Cochrane Library databases. A meta-analysis employing a random-effects model was performed to assess the pooled effects.

**Results:**

The systematic review and meta-analysis included 32 and 18 longitudinal studies, respectively. The prediction of 1- to 2-year all-cause mortality in sarcopenia was not statistically significant (hazard ratio (HR): 1.35, 95% CI 0.76–2.38, *I*^2^ = 54%, *P = *0.31). The lowest combined quartile and quantile of the population were used to define low handgrip strength that showed identical results (HR: 1.24, 95% CI 0.94–1.62, *I*^2^ = 0%, *P = *0.13). Low L3-L4 psoas muscle mass (HR: 2.20, 95% CI 1.26–3.83, *I*^2^ = 87%, *P < *0.01) and slow gait speed (HR: 1.45, 95% CI 1.20–1.74, *I*^2^ = 0%, *P < *0.01) were significant contributors to all-cause mortality risk. Additionally, a 0.1 m/s increase in gait speed demonstrated a significant reduction of all-cause mortality (HR: 0.77, 95% CI 0.66–0.90, I2 = 60%, *P* < 0.01). Our narrative synthesis also described appendicular lean mass (ALM) and short physical performance battery (SPPB) scores as significant prognostic factors.

**Conclusions:**

Compared to patients with higher overall functional performance, those with HF and low ALM, low psoas muscle mass, low SPPB, and slow gait speed are at an increased risk of all-cause mortality. Early prevention and/or treatment of lower limb physical function deterioration may be an essential strategy to reduce the risk of premature death in HF.

**Graphical abstract:**

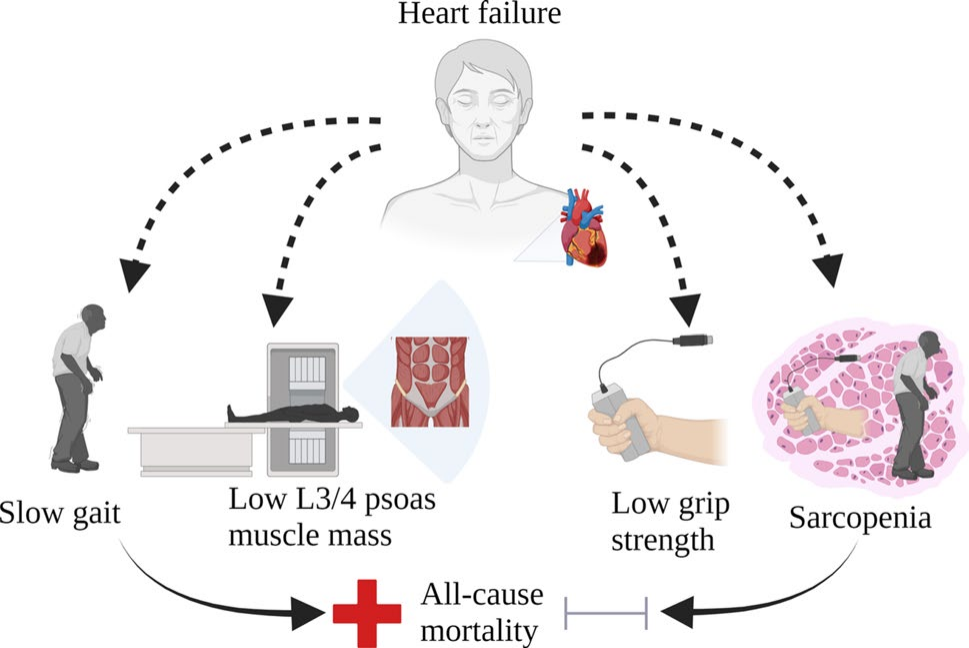

**Supplementary Information:**

The online version contains supplementary material available at 10.1007/s00392-023-02360-8.

## Introduction

Chronic heart failure (CHF) is a cardiovascular disease that is accompanied by an increased risk of morbidity and mortality. The 2019 Heart Failure Association (HFA) ATLAS indicated an HF prevalence ranging from ≤ 12 in Spain and Greece to above > 30 per 1000 people in Lithuania and Germany. In the United States prevalence of HF was evaluated at 2.4%, while in Asia ranged from 1.3% to 6.7% [1]. HF with preserved ejection fraction (HFpEF) (≥ 50% left ventricular ejection fraction (LVEF) and HF with reduced ejection fraction (HFrEF) (< 40% LVEF) are considered the two dominant subgroups of the HF population.

A prominent feature of patients with HF leading to unintentional weight loss is cardiac cachexia, for which, weight loss > 7.5% and/or > 6.0% of body weight has been used as a distinguishing criterion in earlier HF investigations [2, 3]. Given that 2% of the 742 million people in Europe have CHF and that 80% of them are at risk of developing cachexia, it has been assumed that approximately 1.2 million are at risk of cardiac cachexia [4].

Cachexia is accompanied by severe losses of skeletal muscle, contributing to secondary sarcopenia in people with HF [5]. Sarcopenia has been described as the age-related loss of skeletal muscle mass, strength, and physical performance leading to an increased risk of falls, fractures, and loss of independence, although secondary sarcopenia may be also potentiated during HF [6]. Past research has shown that patients with HF have a ~ 20% prevalence of muscle wasting that is accompanied by reductions in handgrip and quadriceps strength, maximal oxygen capacity, and slower walking speed [7]. Recently, using data from 68,556 patients with HF, the prevalence of sarcopenia was estimated at 31% with significant differences among continents, age groups, and ejection fraction rates [8]. Impaired skeletal muscle mitochondrial density, function, and oxidative capability are only a few of the characteristics that have been linked to aggravating muscle loss in patients with HF [9]. Additionally, lower muscle type I to type II ratio and increased intramuscular fat infiltration in this clinical group have also been observed [10]. Although patients with HF are at an increased risk of mortality, the additional burden of sarcopenia may exacerbate the incidence of death. Indeed, a previous meta-analysis showed that low 6-min walking distance was significantly associated with higher mortality risk (mean hazard ratio (HR): 2.04–2.29) vs. those with normal walking distance [11, 12], while similar findings have also been reported regarding slow gait speed (mean HR: 1.49; *P < *0.01) [12]. However, it is worth stating that low and normal gait speed definitions were inconsistent among studies, which partially alleviates the precision of these findings. The aim of this systematic review and meta-analysis is to evaluate the prognostic factor of sarcopenia -as defined by various working groups- and its individual components in patients with HF.

## Methods

This systematic review and meta-analysis were conducted in accordance with the Preferred Reporting Items for Systematic Reviews and Meta-Analyses (PRISMA) guidelines [13]. The protocol was registered in the International Prospective Register of Systematic Reviews (PROSPERO) (CRD42023378427).

### Search strategy

Two independent reviewers (K.P. and K.K.T) searched PubMed, Scopus, Web of Science, and Cochrane Library from inception until December 2022. The full search strategy and the search terms used are described in Table S1. Discrepancies in the literature search process were resolved by a third investigator (M.I.).

### Inclusion and exclusion criteria

Studies were included based on the following criteria: (i) prospective cohort studies; (ii) individuals ≥ 18 years old) with HF irrespective of type; and (iii) assessment of prognostic impact via HRs of a muscle health-related outcome (i.e., sarcopenia, psoas muscle mass, handgrip strength, appendicular lean mass (ALM), gait speed, and short physical performance battery (SPPB)) on all-cause mortality. Published articles were excluded if they (i) were reviews, letters, in vivo or in vitro experiments or commentaries; and (ii) were not published as a full text.

### Data extraction and risk of bias

Two authors (K.P. and K.K.T) extracted data independently, which included the name of the first author, date of publication, country of origin, definition of sarcopenia, sample size and age of participants, type of HF, left ventricular ejection fraction rate (%), outcome of interest, follow-up duration, and muscle mass assessment tool. Disagreements between authors were resolved by two independent reviewers (K.S.K and A.M). The quality of the included studies was evaluated using the Methodological index for non-randomized studies (MINORS) tool [14] and performed by two independent reviewers (K.K.T and K.S.K). MINORS is a comprehensive tool used to assess bias in non-randomized controlled trials based on the following items: a clearly stated aim; inclusion of consecutive patients; prospective data collection; endpoints appropriate to study aim; unbiased assessment of study endpoint; follow-up period appropriate to study aim; < 5% lost to follow-up; prospective calculation of study size; adequate control group; contemporary groups; baseline equivalence of groups, and adequate statistical analyses. According to the scoring system, MINORS’ domains are scored as 0 if they are not reported, 1 when they have been reported but with inadequate details, and 2 when they have been reported while providing adequate information. The global ideal score is 16 for non-comparative studies and a score below 8 was deemed as a high risk of bias and of some concerns, respectively.

### Statistical analysis

The meta-analyses were conducted for each outcome of interest with a minimum of two or more studies, considering the assessment of identical indices of muscle health and measurement units, such as HR. Outcomes of interest that were excluded from the meta-analysis were reported using a narrative synthesis. Statistical significance was assessed using the random effects model and inverse-variance method. Statistical heterogeneity of outcome measurements between different studies was assessed using the overlap of their confidence interval (95% CI) and expressed as measurements of Cochran's Q (Chi-square test) and *I*^2^. The classification of data as having low heterogeneity was based on *I*^2^ from 30 to 49%, moderate heterogeneity from 50 to 74%, and high heterogeneity from 75% and above [15]. Subgroup analyses based on the lowest tertile and quartile of psoas muscle mass, follow-up duration, slow gait speed defined as < 0.8 m/s, and gait speed for each 0.1 m/s increase were performed. Moreover, further analyses were employed to evaluate the robustness of reported statistical results by discounting the effect of identical definitions of slow gait speed (i.e., SGS ratio), and the lowest quartile/tertile outcomes. Additionally, sensitivity analysis was intended to improve the accuracy of our findings by excluding studies conducted in populations with left ventricular assist device therapy (LVADT) (handgrip strength), transcatheter aortic valve implantation and replacement (psoas muscle), and studies with a higher risk of bias. The meta-analysis was synthesized using Review Manager (RevMan 5.4.1) software. A *p* value of < 0.05 was considered statistically significant.

### Definition of outcomes

Sarcopenia was defined based on the European Working Group on Sarcopenia in Older People (EWGSOP1) [16] and the Asian Working Group for Sarcopenia (AWGS) [17].

Information around low ALM was derived from details pertinent to a 1% increase of ALM [18], a 1 kg increase of ALM [19], a cut-off of < 7.0 kg/m^2^ in men and < 5.7 kg/m^2^ in women for low ALM [20], and a cut-off of < 7.26 kg/m^2^ in men and < 5.45 kg/m^2^ in women for low ALM [21].

Regarding low handgrip strength, four studies used the lowest quantile adjusted for gender and body mass index (BMI) [22–25] and one study the lowest quartile [26]. As part of our narrative synthesis, low handgrip strength was defined as < 30 kg in men and < 17.5 kg in women [27], < 32 kg [28], < 26 kg in men and < 18 kg in women [20], < 25.5 kg [29], the lowest quantile adjusted for gender and body mass index (BMI) but with a 30-day all-cause mortality [30], 10.1 kg/m^2^ in men and < 7.95 kg/m^2^ in women [31], and as per 1 kg decrease of handgrip strength [32].

Low (L3-L4) psoas muscle mass was defined as the lowest quartile based on gender in two studies [33, 34] and the lowest tertile based on gender in three studies [35–37]. Finally, in one study, low psoas muscle mass was defined as ≤ 635 mm^2^/m^2^ in men and ≤ 856 mm^2^/m^2^ in women.

Slow gait speed was defined as < 0.8 m/s in two studies [20, 23], a cut-off value of < 0.527 according to a standardized gait speed (SGS) ratio; SGS was defined as the median gait speed stratified by age, sex, and height [38], and the slowest quantile based on time to walk 15 feet, adjusting for gender and standing height in three studies [22, 23, 39]. Moreover, two studies explored all-cause mortality based on a 0.1 m/s gait speed increase [40, 41]. For our narrative synthesis, low gait speed was defined as < 0.98 m/s [27], < 0.83–0.5 and < 0.5 m/s [42], slowest quantile based on time to walk 15 feet, adjusting for gender and standing height but for 30-day all-cause mortality [30] and 8.7-year all-cause mortality [24], slowest quartile [43], and walking 5-m in more than 6 s [25].

Low SPPB was defined as 0 and 1–4 in one study [44] and ≤ 10 in HFrEF, ≤ 9 in HF(medium range)EF, and ≤ 8 in the HFpEF group based on the Youden index in another study [45].

## Results

### Literature search

**Fig. 1:**
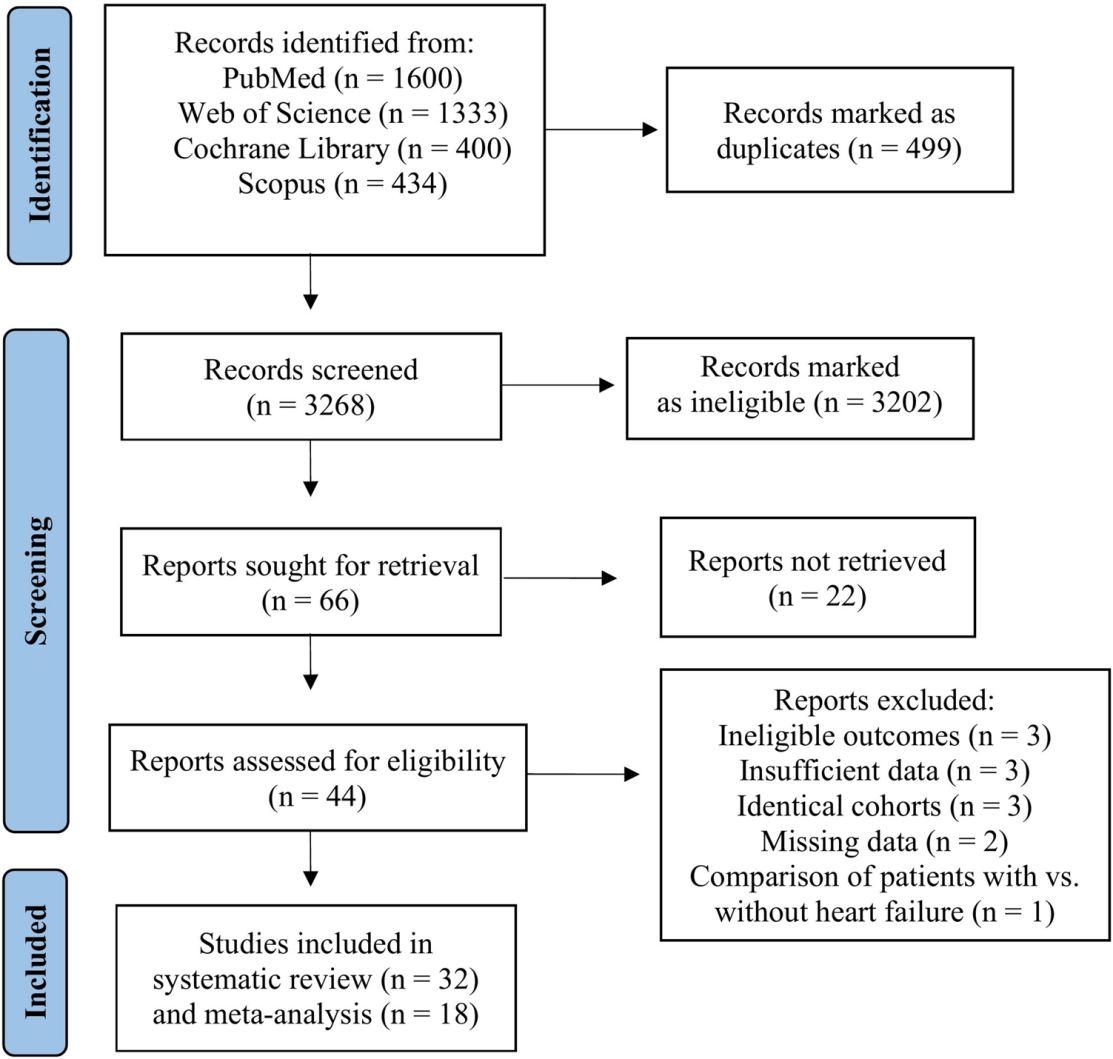
Flowchart of the literature search

The initial literature search provided 3767 publications. Following the exclusion of duplicates (*n = *499), 3268 abstracts and full texts were screened from which 3202 were marked as ineligible. Of the remaining 66 studies, 22 studies were not retrieved and eventually, 44 reports were assessed for eligibility. Of these 44 studies, three studies were dismissed due to ineligible outcomes, three studies due to insufficient data, three studies due to identical cohorts with more recent studies included in the systematic review and/or meta-analysis, two studies had missing data and one study that compared patients with vs. without HF. In total, 32 studies were included in the systematic review and 18 studies in the meta-analysis (Fig. 1). Characteristics of the included studies are detailed in Tables S2–S6.

### Sarcopenia and all-cause mortality

**Fig. 2:**
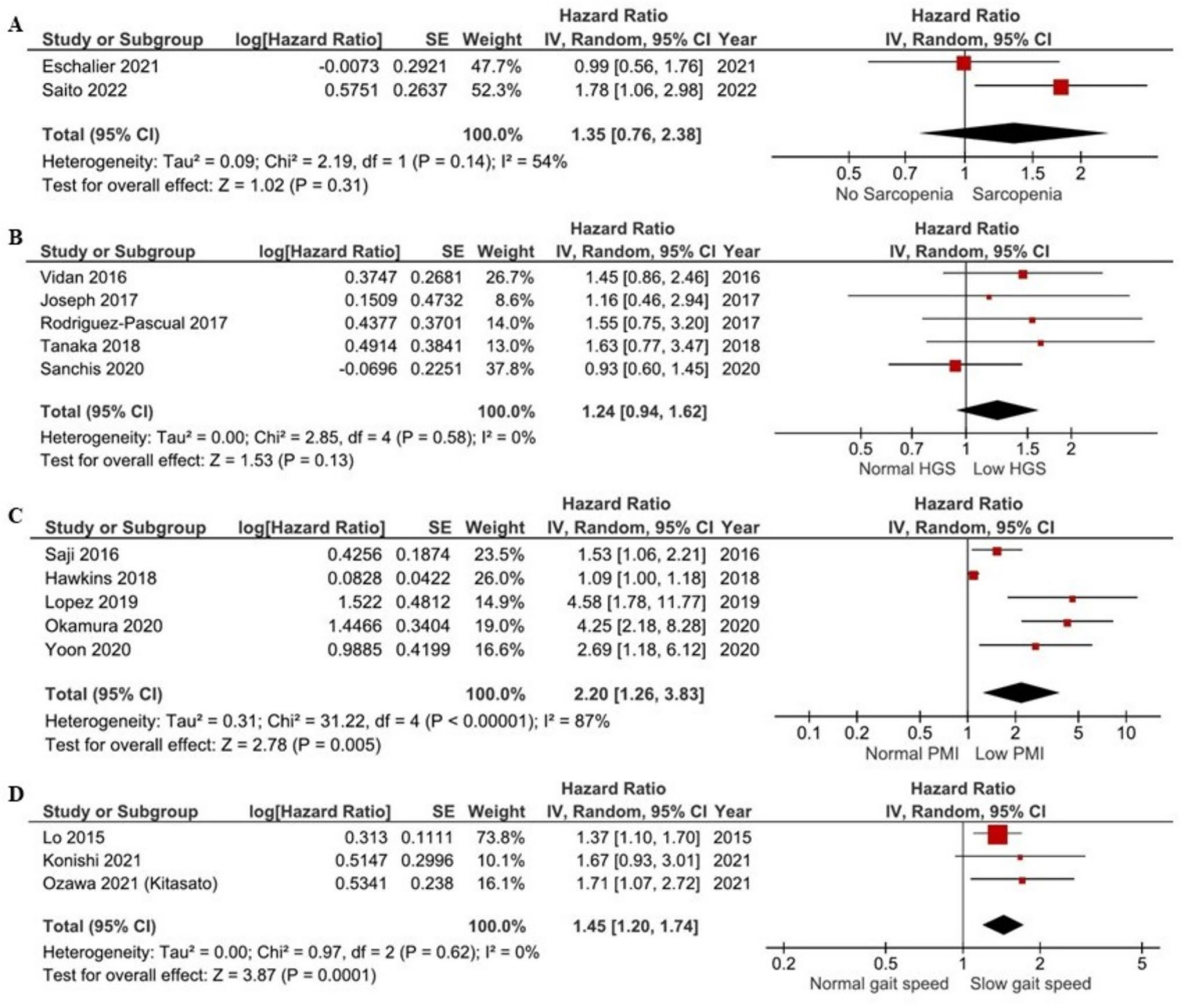
Effects of sarcopenia (**A**), low handgrip strength (HGS) (lowest quartile and quantile combined) (**B**), low L3-L4 psoas muscle index (PMI) (low tertiles and quartiles combined) (**C**), and slow gait speed (**D**) on one-to-two-year all-cause mortality in patients with HF

Regarding sarcopenia, the prognosis of 1-to-2-year all-cause mortality did not reach statistical significance with moderate heterogeneity between studies (*k* = 2; HR: 1.35, 95% CI 0.76–2.38, *I*^2^ = 54%, *P = *0.31; Fig. 2A).

### Low appendicular lean mass and all-cause mortality

ALM seemed to be a significant prognostic factor of all-cause mortality in patients with HF in two studies that examined HFpEF and HFrEF separately. Particularly, Konishi et al*.* (2021b) [20] found a HR of 2.46 (95% CI 1.39–4.37, *P < *0.01) for 1-year mortality in HFpEF patients with low ALM, while von Haehling et al*.* (2020) [21] demonstrated identical findings in only the HFrEF phenotype (Overall–HR: 1.80, 95% CI 1.01–3.19, *P = *0.04; HFrEF–HR: 1.97, 95% CI 1.05–3.71, *P = *0.04; HFpEF–HR: 1.86, 95% CI 0.32–10.70, *P = *0.49). Interestingly, Katano and colleagues [18] found that per 1.0% increase in ALM, all-cause mortality was significantly reduced (HR: 0.979, 95% CI 0.961–0.998, *P = *0.02). Finally, one study [19] also reported a significant reduction of all-cause mortality per 1 kg of ALM increase (HR: 0.84, 95% CI 0.76–0.93, *P < *0.01).

### Low handgrip strength and all-cause mortality

Our analysis showed that low handgrip strength (lowest quartile and quantile combined) was not a prognostic factor of all-cause mortality (*k* = 5; HR: 1.24, 95% CI 0.94–1.62, *I*^2^ = 0%, *P = *0.13) (Fig. 2B). Similarly, when we excluded one study to evaluate the prognostic factor of those with handgrip strength in the lowest quartile no changes were observed (HR: 1.19, 95% CI 0.89–1.58, *I*^2^ = 0%, *P = *0.25) (Fig. S1). Given that the study by Sanchis et al*.* (2020) [24] evaluated 8.7-year all-cause mortality, in a sensitivity analysis including studies with 1-to-3 years of all-cause mortality as the final outcome, we found that those in the lowest quartile and quantile of handgrip strength combined had a statistically significant chance of dying of any cause sooner (HR: 1.47, 95% CI 1.04–2.07, *I*^2^ = 0%, *P = *0.03) (Fig. S2). Finally, in another sensitivity analysis we excluded one study in which participants were under LVADT, however, no significant changes were observed (HR: 1.24, 95% CI 0.94–1.65, *I*^2^ = 0%, *P = *0.13) (Fig. S3).

In our narrative synthesis, handgrip strength was deemed a significant prognostic factor for all-cause mortality both in the short and the long term. Two studies that investigated its prognosis within a 1 to 2-year period found significant changes [20]; HFrEF, HR: 2.55, 95% CI 1.27–5.10, *P < *0.01; HFpEF, HR: 2.23, 95% CI 1.00–5.14, *P < *0.05)–[27]; HR: 1.95, 95% CI 0.99–3.83, *P < *0.05), however, one study [31] revealed no differences (3-year mortality) (Men; HR: 0.93, 95% CI 0.37–2.30, *P = *0.8–Women; HR: 1.5, 95% CI 0.52–4.1, *P = *0.48). Additionally, those with increased handgrip strength (≥ 32.2 kg had improved survival rates (44.3 months median follow-up) (HR: 0.9, 95% CI 0.83–0.98, *P < *0.01) [28]. In the short-term, low handgrip strength (< 25.5 kg) was also a significant contributor to all-cause mortality (90 days [29]: HR: 8.6, 95% CI 1.1–70.9, *P = *0.045) (30 days [30]: HR: 2.4, 95% CI 1.0–5.9).

### Low psoas muscle mass and all-cause mortality

Our analysis showed that low L3-L4 PMI (low tertiles and quartiles combined) was a significant prognostic factor of all-cause mortality in patients with HF (*k* = 5; HR: 2.20, 95% CI 1.26–3.83, *I*^2^ = 87%, *P < *0.01) (Fig. 2C), however, a high heterogeneity was observed. When we categorised our groups to very long-term (5–5.5 years) and shorter-term (6–12 months) mortality, our analyses showed only a prognostic impact of low PMI within 1 year (HR: 2.38, 95% CI 1.24–4.58, *I*^2^ = 63%, *P < *0.01) (Fig. S4) (5–5.5 years all-cause mortality; HR: 2.06, 95% CI 0.54–7.82, *I*^2^ = 94%, *P = *0.29) (Fig. S5). Regarding short-term (30 days) mortality, one study found that low P3 PMI demonstrated substantial risk of death (HR: 27.3, 95% CI 2.74–272.797, *P < *0.01).

### Slow gait speed and all-cause mortality

Our analysis revealed that slow gait speed was a significant prognostic factor of 1-year all-cause mortality (*k* = 3; HR: 1.45, 95% CI 1.20–1.74, *I*^2^ = 0%, *P < *0.01) (Fig. 2D). Given that the study by Ozawa et al*.* (2021) [38] created their own definition of slow gait speed based on the values (< 0.8 m/s) derived by community-dwelling older adults, a sensitivity analysis was performed, although results remained significant (HR: 1.40, 95% CI 1.14–1.72, *I*^2^ = 0%, *P < *0.01) (Fig. S6). In addition, we performed an additional analysis according to the HFpEF phenotype and slow gait speed (< 0.8 m/s) in which we found significant outcomes (HR: 1.48, 95% CI 1.17–1.86, *I*^2^ = 0%, *P < *0.01) (Fig. S7). When we calculated all-cause mortality risk in those with the slowest quantile [39], we also found a significant change (HR: 1.53, 95% CI 1.05–2.23, *I*^2^ = 0%, *P = *0.03) (Fig. S8). Considering that the study be Zheng et al*.* (2021) had a moderate risk of bias, our sensitivity analysis showed an even higher risk of mortality (HR: 1.81, 95% CI 1.15–2.86, *I*^2^ = 0%, *P = *0.01) (Fig. S9). Furthermore, we attempted to examine whether improvements in gait speed would correspond in lower all-cause mortality rates. For each 0.1 m/s increase in gait speed, meta-analysis demonstrated a significant reduction of all-cause mortality (HR: 0.77, 95% CI 0.66–0.90, *I*^2^ = 60%, *P < *0.01) (Fig. S10), although one study assessed prognosis at 2.1 years and the other at 5.5 years.

Our narrative synthesis revealed mixed results in relation to slow gait speed being a significant prognostic factor of all-cause mortality. Specifically, the slowest quintile within was a contributor to long-term mortality (8.7 years median follow-up) (HR: 2.21, 95% CI 1.34–3.65, *P < *0.01) [24]. Using quartiles, no changes were observed with the slowest group (< 0.82 m/s) (HR: 1.38, 95% CI 0.72–2.62, *P = *0.33), however, the highest quartile in another study showed a greater survival rate (HR: 0.2, 95% CI 0.07–0.56, *P < *0.01) [43]. Interestingly, a similar slow gait speed group (0.5–0.83 m/s) also showed no prognostic impact for 1-year all-cause mortality (HR: 1.06, 95% CI 0.66–1.68, *P = *0.82), however, the slowest group in this study (< 0.5 m/s) had a substantial contribution (HR: 2.14, 95% CI 1.32–3.46, *P < *0.01) [42]. When cut-offs increased substantially (slow gait speed at < 0.98 m/s), no significant changes were observed pertinent to 2-year all-cause mortality (HR: 1.32, 95% CI 0.66–2.65, *P = *0.44) [27], suggesting that very low values of gait speed may be a significant contributor to a higher mortality rate in this population. In another study [30], although HR values were deemed high, p values were not reported for 30-day mortality (HR: 4.1, 95% CI 1.5–10.8). Finally, walking a 5-m distance in more than 6 s, was not a prognostic factor for 3-year mortality (HR: 1.05, 95% CI 0.36–3.09, *P = *0.9) [25].

### Low short physical performance battery and all-cause mortality

Low SPPB scores (score = 0, 1–4) were a significant prognostic determinant of 30-month all-cause mortality in patients with HF (0 score; HR: 6.06, 95% CI 2.19–16.76, *P < *0.01–1–4 score; HR: 4.78, 95% CI 1.63–14.02, *P < *0.01), which was not the case with moderate-to-high scores (5–8) (HR: 1.95, 95% CI 0.67–5.70, *P = *0.22) [44]. High SPPB performance (≥ 8) may reduce mortality risk within 2.5 years (HR: 0.87, 95% CI 0.84–0.91, *P < *0.01), while based on the Youden index (≤ 10 in HFrEF, and ≤ 8 in the HFpEF group), low SPPB score has a predictive value for 2-year all-cause mortality in both HFrEF and HFpEF phenotypes (HFrEF; HR: 5.38, 95% CI 2.34–14.6, *P < *0.01–HFpEF; HR: 3.19, 95% CI 1.69–6.22, *P < *0.01) [45].

### Risk of bias of the included studies

Although five of the included studies were deemed to have some concerns pertinent to the risk of bias [30, 33, 39, 42, 46], risk of bias from the rest of the studies was deemed low (Table S7).

## Discussion

In this systematic review and meta-analysis of 32 studies, we found that patients with HF and low ALM and psoas muscle mass, slow gait, and low SPPB, have a greater risk of primarily 1- to 2-year all-cause mortality compared to patients with normal values in these components. Although we found a higher risk of mortality due to sarcopenia and low handgrip strength, our analyses did not reveal statistical significance. Finally, improvements in gait speed were linked to a significantly greater chance of survival.

Similar to our findings, Yamamoto et al*.* (2020) [11] demonstrated significant improvements for every one-meter increase in the 6-min walking distance, supporting our findings pertinent to gait speed improvements. It is worth noting that Fuentes-Abolafio et al*.* (2020) [12] also performed an analysis based on the prognostic factor of slow gait speed. However, there was inconsistency regarding the definition of lower gait speed given that the included studies of Rodriguez-Pascual et al*.* (2017) [22] used the slowest 20% of the population that was defined at baseline, based on time to walking 15-feet after adjusting for gender and standing height, while Tanaka et al*.* (2018) [26] used a < 0.82 m/s cut-off value. Added to this, there seemed to be a typo in the authors’ analysis, considering that in the included study by Vidan et al*.* (2016) [23] the authors used the HR values derived via weight loss instead of slow walking. Finally, low values in SPPB that captures pivotal components of lower limb function was also a significant contributor to increased risk of mortality albeit using data from two studies [44, 45]. Considering the different definitions of SPPB, a meta-analysis could not be performed.

In relation to handgrip strength, the majority of studies depicted a non-significant link with mortality, whilst all four studies that measured the prognostic impact of low ALM showed a statistically significant hazard risk. These deviations could partially explain the non-significant outcomes of sarcopenia, particularly the results shown by Eschalier et al*.* (2021) which revealed a mean HR of 1 [47]. The aforementioned findings should not only encourage primary importance of gait speed as a surrogate marker of lower limb function, but to also strengthen the research and clinical application of the identification and prevention of relevant causes, considering the detrimental impact of falls and fractures on gait, quality of life, and survival [48].

### Strengths and limitations

The strength of this study is the examination of multiple indices of sarcopenia on all-cause mortality rate in patients with HF, combining multiple outcomes with identical definitions through studies with relatively low risk of bias. Our study, however, was prone to several limitations. First, we could not extrapolate findings based on sex and ejection fraction rates, particularly different HF phenotypes (i.e., HFrEF vs. HFpEF). In addition, although the majority of the included studies used multivariate analysis to delve into the relationship between sarcopenia and its components with mortality risk in patients with HF, different adjustments for confounders were made among studies, which could have altered our findings. Furthermore, some studies could not be retrieved due to different languages, full-text access issues, and incomplete reporting, introducing bias. Lastly, we could not perform a meta-analysis on SPPB and ASM to quantify our results and due to lack of uniformity, a limited number of studies were utilized in our meta-analyses.

## Conclusions

Patients with HF accompanied by low ALM, psoas muscle mass, SPPB score, and slow gait speed are at a significantly greater risk of mortality compared to patients who report higher functional performance. Higher consistency in measures of sarcopenia could reveal more accurate and quantifiable findings in this population group. Early diagnosis of sarcopenia in clinical practice, especially of physical deterioration of the lower limbs such as slow gait speed, is of critical importance leading to an earlier therapeutic decision and deserves further investigation.

## Supplementary Information

Below is the link to the electronic supplementary material.

Supplementary file1 Figure S1. Effects of low handgrip strength on all-cause mortality after exclusion of participants with handgrip strength in the lowest quartile. (JPG 251 kb)

Supplementary file2 Figure S2. Effect of the lowest quartile and quantile of handgrip strength combined on all-cause mortality in patients with HF. (JPG 252 kb)

Supplementary file3 Figure S3. Effects of low handgrip strength on all-cause mortality in patients with HF after exclusion of participants undergoing LVADT. (JPG 246 kb)

Supplementary file4 Figure S4. Effects of low L3-L4 PMI (low tertiles and quartiles combined) on 6 to 12-month all-cause mortality in patients with HF. (JPG 249 kb)

Supplementary file5 Figure S5. Effects of low L3-L4 PMI (low tertiles and quartiles combined) on 5 to 5.5-year all-cause mortality in patients with HF. (JPG 251 kb)

Supplementary file6 Figure S6. Effects of slow gait speed on all-cause mortality in patients with HF after exclusion of different definitions of slow gait speed. (JPG 194 kb)

Supplementary file7 Figure S7. Effects of slow gait speed on all-cause mortality in patients with HF based on a higher number of participants with HFpEF. (JPG 174 kb)

Supplementary file8 Figure S8. Effects of slow gait speed (slowest quantile) on all-cause mortality in patients with HF. (JPG 211 kb)

Supplementary file9 Figure S9. Effects of slow gait speed on all-cause mortality in patients with HF based on RoB assessment. (JPG 209 kb)

Supplementary file10 Figure S10. Effects of increased (per 0.1 m/s) gait speed on all-cause mortality in patients with HF. (JPG 171 kb)

Supplementary file11 (DOCX 14 kb)

Supplementary file12 (DOCX 18 kb)

Supplementary file13 (DOCX 17 kb)

Supplementary file14 (DOCX 16 kb)

Supplementary file15 (DOCX 18 kb)

Supplementary file16 (DOCX 14 kb)

Supplementary file17 (DOCX 26 kb)

## Data Availability

All materials used in this review are publicly available. Restrictions of materials due to journals’ different policies may apply, therefore, the authors are happy to provide any data that readers cannot access.
