# Supplementary figures and images for: Are sarcopenia and its individual components linked to all-cause mortality in heart failure? A systematic review and meta-analysis
Source: Clin Res Cardiol. 2023 Dec 12;114(5):532–40. doi: 10.1007/s00392-023-02360-8 (PMC12058882; doi:10.1007/s00392-023-02360-8)

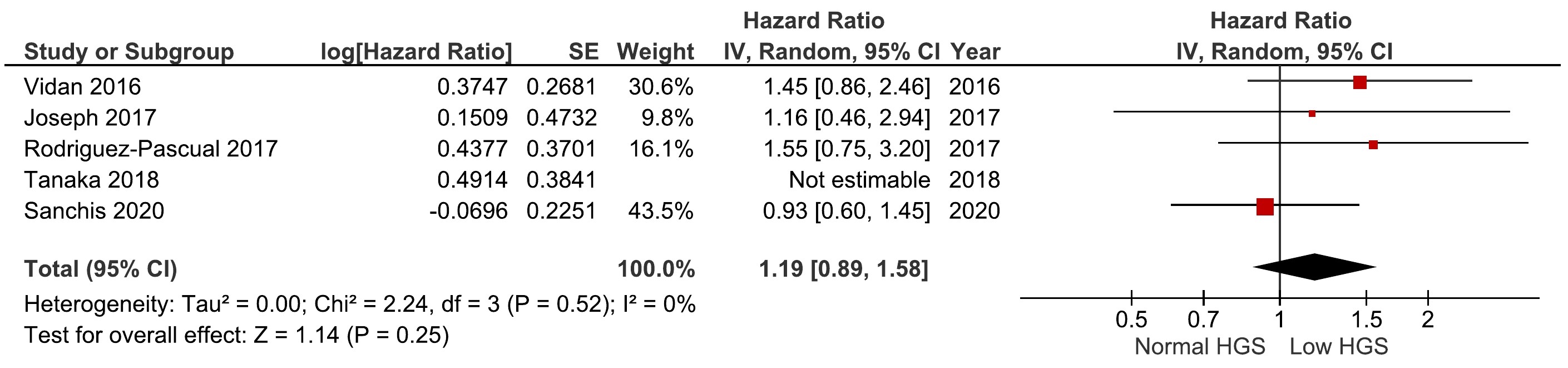

Supplement: Supplementary file 1 — Supplementary file1 Figure S1. Effects of low handgrip strength on all-cause mortality after exclusion of participants with handgrip strength in the lowest quartile. (JPG 251 kb) [file 392_2023_2360_MOESM1_ESM.jpg]

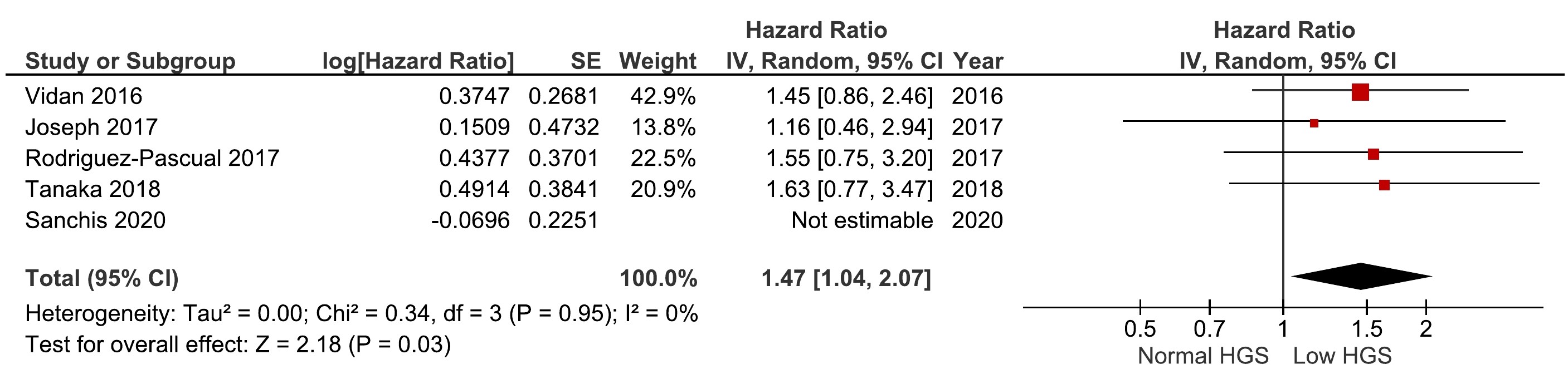

Supplement: Supplementary file 2 — Supplementary file2 Figure S2. Effect of the lowest quartile and quantile of handgrip strength combined on all-cause mortality in patients with HF. (JPG 252 kb) [file 392_2023_2360_MOESM2_ESM.jpg]

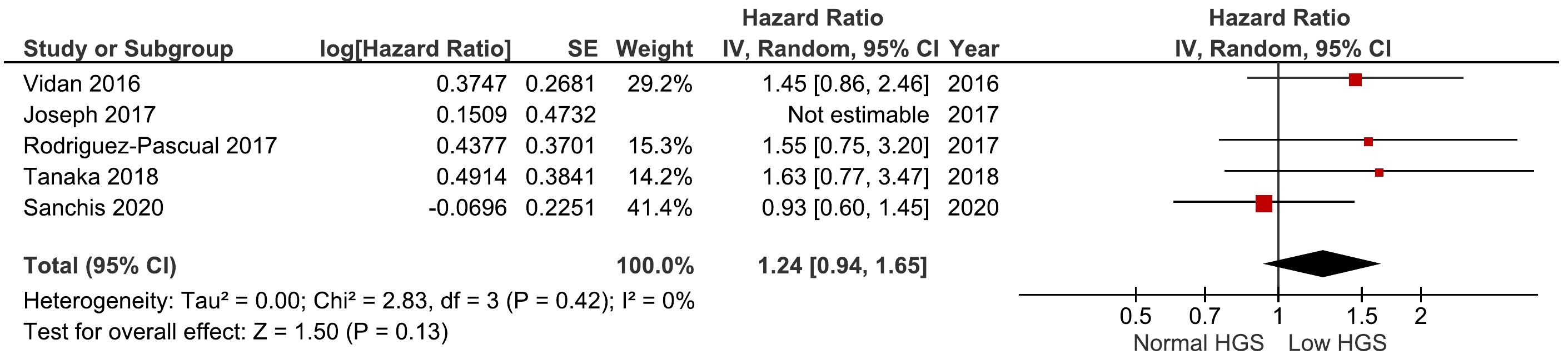

Supplement: Supplementary file 3 — Supplementary file3 Figure S3. Effects of low handgrip strength on all-cause mortality in patients with HF after exclusion of participants undergoing LVADT. (JPG 246 kb) [file 392_2023_2360_MOESM3_ESM.jpg]

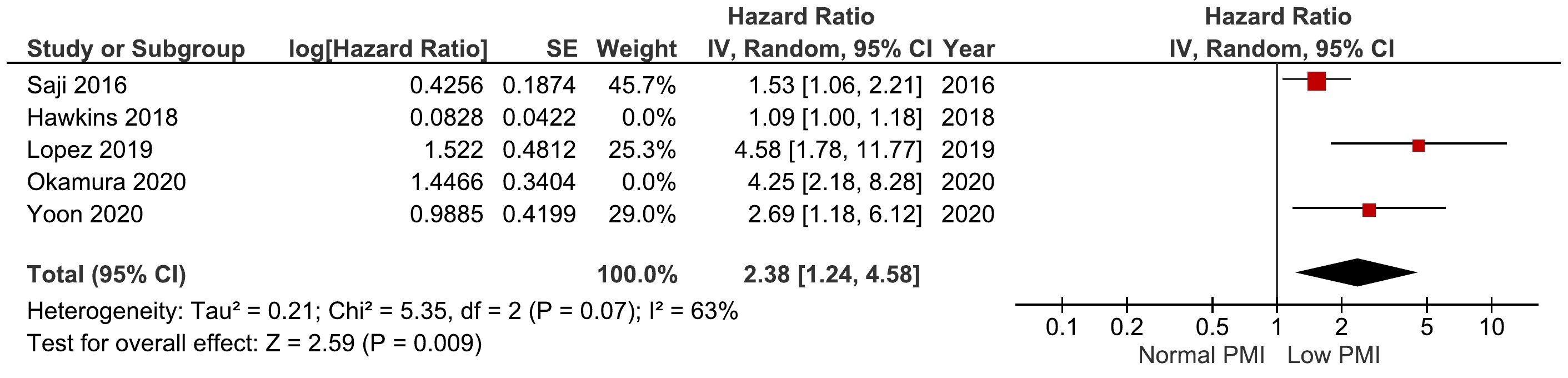

Supplement: Supplementary file 4 — Supplementary file4 Figure S4. Effects of low L3-L4 PMI (low tertiles and quartiles combined) on 6 to 12-month all-cause mortality in patients with HF. (JPG 249 kb) [file 392_2023_2360_MOESM4_ESM.jpg]

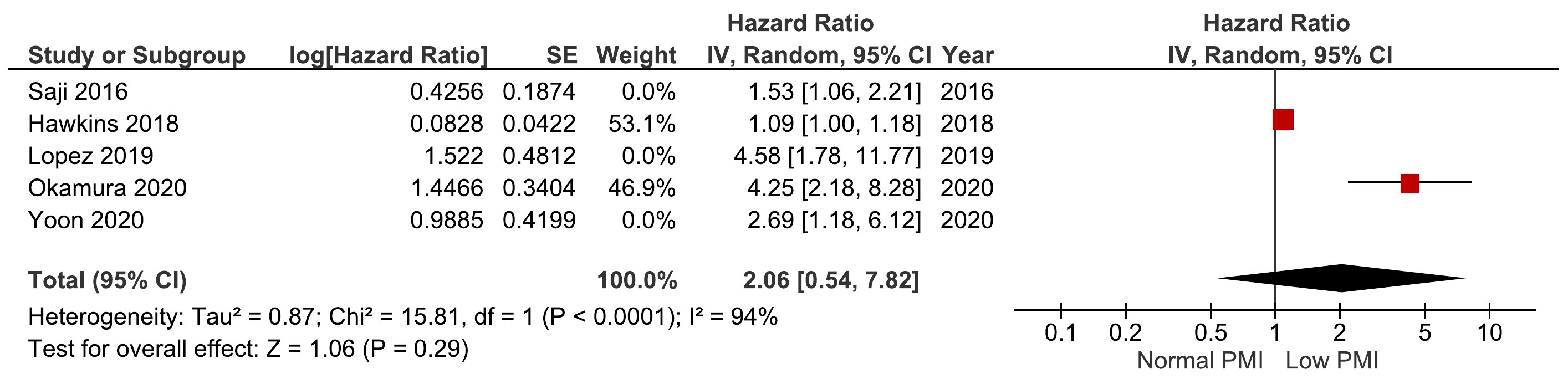

Supplement: Supplementary file 5 — Supplementary file5 Figure S5. Effects of low L3-L4 PMI (low tertiles and quartiles combined) on 5 to 5.5-year all-cause mortality in patients with HF. (JPG 251 kb) [file 392_2023_2360_MOESM5_ESM.jpg]

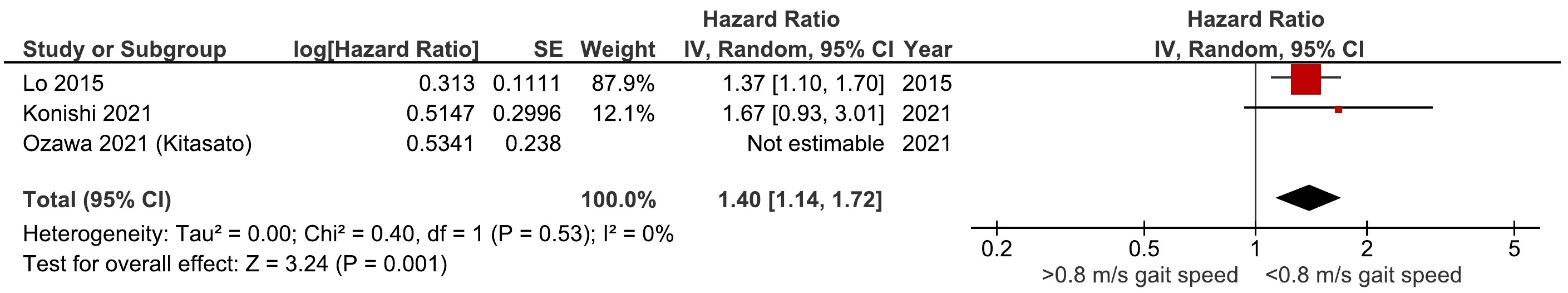

Supplement: Supplementary file 6 — Supplementary file6 Figure S6. Effects of slow gait speed on all-cause mortality in patients with HF after exclusion of different definitions of slow gait speed. (JPG 194 kb) [file 392_2023_2360_MOESM6_ESM.jpg]

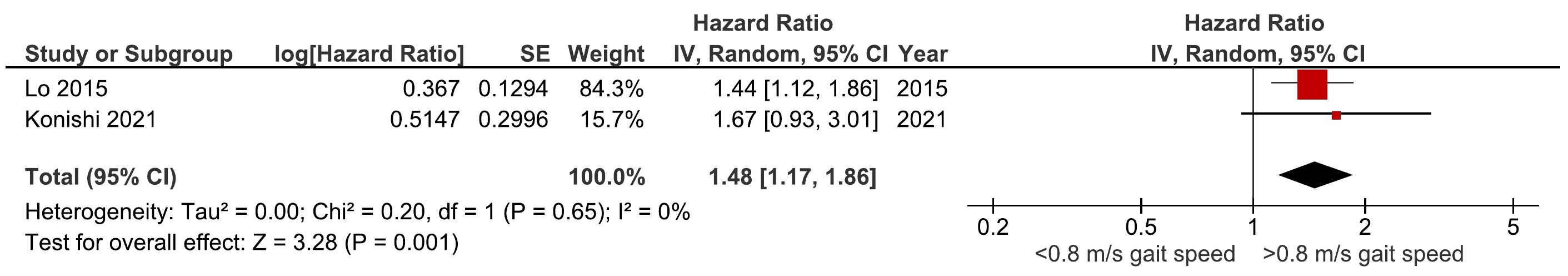

Supplement: Supplementary file 7 — Supplementary file7 Figure S7. Effects of slow gait speed on all-cause mortality in patients with HF based on a higher number of participants with HFpEF. (JPG 174 kb) [file 392_2023_2360_MOESM7_ESM.jpg]

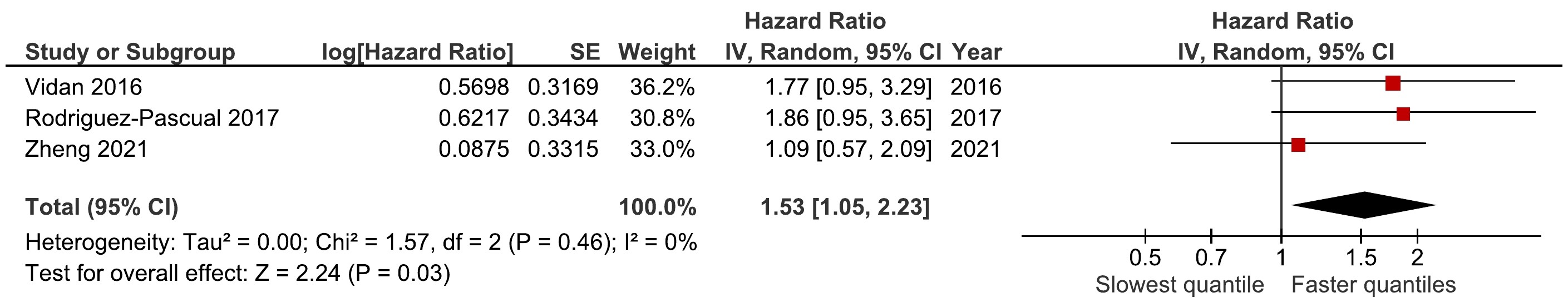

Supplement: Supplementary file 8 — Supplementary file8 Figure S8. Effects of slow gait speed (slowest quantile) on all-cause mortality in patients with HF. (JPG 211 kb) [file 392_2023_2360_MOESM8_ESM.jpg]

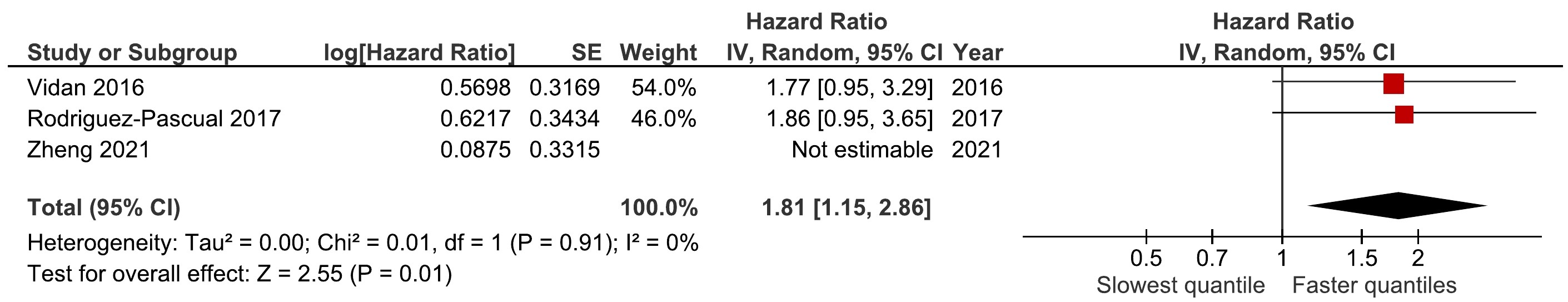

Supplement: Supplementary file 9 — Supplementary file9 Figure S9. Effects of slow gait speed on all-cause mortality in patients with HF based on RoB assessment. (JPG 209 kb) [file 392_2023_2360_MOESM9_ESM.jpg]

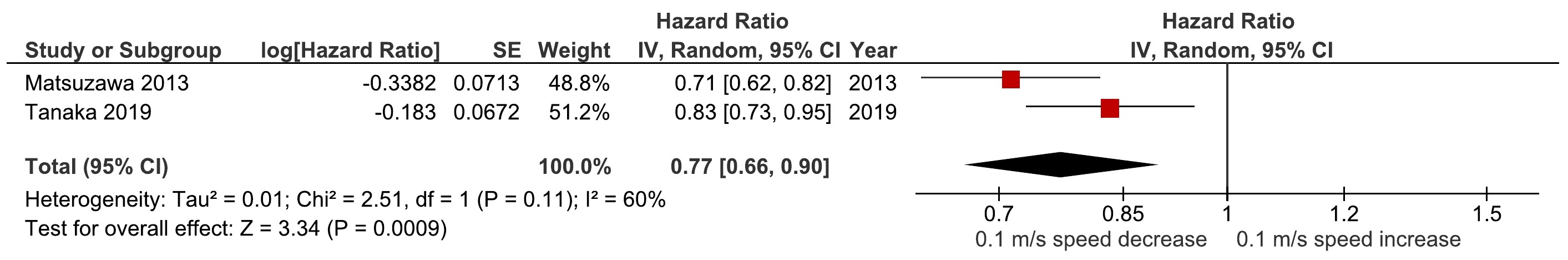

Supplement: Supplementary file 10 — Supplementary file10 Figure S10. Effects of increased (per 0.1 m/s) gait speed on all-cause mortality in patients with HF. (JPG 171 kb) [file 392_2023_2360_MOESM10_ESM.jpg]
