# Supplementary material for: Are sarcopenia and its individual components linked to all-cause mortality in heart failure? A systematic review and meta-analysis
Source: Clin Res Cardiol. 2023 Dec 12;114(5):532–40. doi: 10.1007/s00392-023-02360-8 (PMC12058882; doi:10.1007/s00392-023-02360-8)
Supplement: Supplementary file 11 — Supplementary file11 (DOCX 14 kb) [file 392_2023_2360_MOESM11_ESM.docx]

**Table S1.**Search terms employed in the literature search.

| **Database** | **Search terms** |
| --- | --- |
|  |  |
| PubMed | (Heart failure OR ejection fraction OR myocardial infarction) AND (sarcopenia OR gait speed OR handgrip strength OR time up to go OR TUG OR SPPB OR short physical performance battery OR muscle mass OR appendicular lean mass OR frail*) AND (mortality OR survival OR death) |
| Cochrane Library | (Heart failure OR ejection fraction OR myocardial infarction) AND (sarcopenia OR gait speed OR handgrip strength OR time up to go OR TUG OR SPPB OR short physical performance battery OR muscle mass OR appendicular lean mass OR frail*) AND (mortality OR survival OR death) |
| Web of Science | (Heart failure OR ejection fraction OR myocardial infarction) AND (sarcopenia OR gait speed OR handgrip strength OR time up to go OR TUG OR SPPB OR short physical performance battery OR muscle mass OR appendicular lean mass OR frail*) AND (mortality OR survival OR death) |
| Scopus | heart AND failure OR ejection OR fraction OR myocardial AND mortality OR survival OR death AND sarcopenia AND frailty |
