# Supplementary material for: Are sarcopenia and its individual components linked to all-cause mortality in heart failure? A systematic review and meta-analysis
Source: Clin Res Cardiol. 2023 Dec 12;114(5):532–40. doi: 10.1007/s00392-023-02360-8 (PMC12058882; doi:10.1007/s00392-023-02360-8)
Supplement: Supplementary file 12 — Supplementary file12 (DOCX 18 kb) [file 392_2023_2360_MOESM12_ESM.docx]

**Table S2.** Study and participant characteristics of the included studies in the systematic review and meta-analysis examining the effect of sarcopenia and low appendicular lean mass on all-cause mortality.

| **Study**  **Year**  **Country** | **Sarcopenia or muscle dysfunction definition** | **Total *n*  (M/F)** | **Patients with sarcopenia or muscle dysfunction definition** | | | **Patients without sarcopenia or muscle dysfunction definition** | | | **Median**  **Follow-up**  **(years)** | **Body Composition Assessment Tool** |
| --- | --- | --- | --- | --- | --- | --- | --- | --- | --- | --- |
|  |  |  | ***n* (M/F)** | **Age**  **(SD)** | **LVEF**  **(%)** | **n**  **(M/F)** | **Age**  **(SD)** | **LVEF**  **(%)** |  |  |
| Saito  2022  Japan | AWGS 2014 | 575  (319/256) | 119  (81/38) | 82 (76-86) | 45 ± 18 | 456 (238/218) | 81 (74-86) | 46 ± 16 | 1 | BIA |
| Eschalier  2021  France | EWGSOP1 | 140  (82/58) | 91  (54/37) | 78.2 ±   9.0 | 42.8 ± 14.7 | 49  (28/21) | 71.4 ± 10.9 | 40.7 ± 14 | 2 | BIA |
| Konishi 2021b  Japan | ALM (<7.0 kg/m^2^ in males and <5.7 kg/m^2^ in females) | 942  (550/392) | 187  (132/55) | 80-86 | HFrEF: 30 ± 7  HFpEF: 61 ±10 | 755  (418/337) | 77-80 | HFrEF: 32 ± 8  HFpEF: 59 ± 9 | 1 | BIA |
| Katano  2022  Japan | ALM  (per 1.0% increase of the mean of ALM) | 539  (307/232) | 335  (201/134) | 73 (median)  66-81 (age range) | 45.9 (median)  32.4-62 (range) | 204  (106/98) | 72 (median)  60-82 (age range) | 51.7 (median)  36.2-64 (range) | 1.75 | DXA |
| Konishi  2021a  Japan | ALM  (per 1kg increase) | 418  (247/171) | - | - | - | - | - | - | 3.1 | DXA |
| von Haehling 2020  Germany | ALM (<7.26 kg/m^2^ in males and <5.45 kg/m^2^ in females) | 268  (211/57) | 47  (45/2) | 79.94 ± 8.6 | - | 221  (166/55) | 66.12 ± 11.04 | - | 5.6 (mean) | DXA |

ALM, appendicular skeletal muscle; AWGS, Asian Working Group for Sarcopenia; BIA, bioelectrical impedance; DXA, dual x-ray absorptiometry; EWGSOP, European Working Group on Sarcopenia in Older People; F, females; HFmrEF; heart failure with mid-range ejection fraction; HFpEF, heart failure with preserved ejection fraction; HFrEF, heart failure with reduced ejection fraction; LVEF, left ventricular ejection fraction; M, males; SD, standard deviation.

Data are expressed as mean ± SD.

Data are expressed as median (IQR).
