# Supplementary material for: Are sarcopenia and its individual components linked to all-cause mortality in heart failure? A systematic review and meta-analysis
Source: Clin Res Cardiol. 2023 Dec 12;114(5):532–40. doi: 10.1007/s00392-023-02360-8 (PMC12058882; doi:10.1007/s00392-023-02360-8)
Supplement: Supplementary file 13 — Supplementary file13 (DOCX 17 kb) [file 392_2023_2360_MOESM13_ESM.docx]

**Table S3.** Study and participant characteristics of the included studies in the systematic review and meta-analysis examining the effect of low handgrip strength on all-cause mortality.

| **Study**  **Year**  **Country** | **Low handgrip strength (HGS) definition** | **Total *n*  (M/F)** | **Patients with low handgrip strength** | | | **Patients without low handgrip strength** | | | **Median**  **Follow-up**  **(years)** |
| --- | --- | --- | --- | --- | --- | --- | --- | --- | --- |
|  |  |  | ***n* (M/F)** | **Age**  **(SD)** | **LVEF**  **(%)** | **n**  **(M/F)** | **Age**  **(SD)** | **LVEF**  **(%)** |  |
| Rodriguez-Pascual  2017  Spain | HGS (lowest 20% at baseline, adjusted for gender and BMI) | 497  (194/303) | - | - | - | - | - | - | 1 |
| Vidán  2016  Spain | HGS (lowest 20% at baseline, adjusted for gender and BMI) | 416  (210/206) | - | - | - | - | - | - | 1 |
| Sanchis  2020  Spain | HGS (lowest 20% at baseline, adjusted for gender and BMI) | 342  (196/146) | - | - | - | - | - | - | 8.7 |
| Tanaka  2018  Japan | HGS quartiles (<20.7, 20.7-25, 25.1-30.4, >30.4 in males) (<13.1, 13.1-16, 16.1-19.4, >19.4 in females) adjusted for gender and BMI | 603  (378/225) | - | - | - | - | - | - | 1.7 (mean) |
| Joseph  2017  USA | HGS (lowest 20% at baseline, adjusted for gender and BMI) | 75  (56/19) | - | - | - | - | - | - | 3 |
| Ashikawa  2022  Japan | HGS (<30 kg in males and <17.5 kg in females) | 489  (354/135) | - | - | - | - | - | - | 2 |
| Sze  2022  United Kingdom | HGS (per 1 kg decrease) | 467  (313/154) | - | - | - | - | - | - | 1 |
| Konishi  2021b  Japan | HGS (<26 kg in males and <18 kg in females) | 942  (550/392) | 187  (132/55) | 80-86 | HFrEF: 30 ± 7  HFpEF: 61 ±10 | 755  (418/337) | 77-80 | HFrEF: 32 ± 8  HFpEF: 59 ± 9 | 1 |
| Izawa  2009  Japan | HGS (<32 kg) | 148  (148/0) | - | - | - | - | - | - | 3.7 |
| Parahiba 2021  Brazil | HGS (<25.5 kg) | 161  (100/61) | - | - | - | - | - | - | 90 days |
| Martin-Sánchez 2017  Spain | HGS (lowest 20% at baseline, adjusted for gender and BMI) | 465  (182/283) | - | - | - | - | - | - | 30 days |
| Castillo-Martínez 2020  Mexico | HGS (<10.1 kg/m^2^ in males and <7.95 kg/m^2^ in females) | 546  (255/246) | 164 | M: 64.9 ± 16.3  F: 63.4 ± 16.9 | M: 44 ± 17  F: 47 ± 17 | 172 | M: 56.6 ± 15.5  F: 55.7 ± 17.8 | M: 45 ± 15  F: 49 ± 16 | 3 years |

BMI, body mass index; F, females; HFmrEF; heart failure with mid-range ejection fraction; HFpEF, heart failure with preserved ejection fraction; HFrEF, heart failure with reduced ejection fraction; LVEF, left ventricular ejection fraction; M, males; SD, standard deviation.

Data are expressed as mean ± SD.

Data are expressed as median (IQR).
