# Supplementary material for: Are sarcopenia and its individual components linked to all-cause mortality in heart failure? A systematic review and meta-analysis
Source: Clin Res Cardiol. 2023 Dec 12;114(5):532–40. doi: 10.1007/s00392-023-02360-8 (PMC12058882; doi:10.1007/s00392-023-02360-8)
Supplement: Supplementary file 14 — Supplementary file14 (DOCX 16 kb) [file 392_2023_2360_MOESM14_ESM.docx]

**Table S4.** Study and participant characteristics of the included studies in the systematic review and meta-analysis examining the effect of low psoas muscle mass on all-cause mortality.

| **Study**  **Year**  **Country** | **Sarcopenia or muscle dysfunction definition** | **Total *n*  (M/F)** | **Patients with sarcopenia or muscle dysfunction definition** | | | **Patients without sarcopenia or muscle dysfunction definition** | | | **Median**  **Follow-up**  **(years)** | **Body Composition Assessment Tool** |
| --- | --- | --- | --- | --- | --- | --- | --- | --- | --- | --- |
|  |  |  | ***n* (M/F)** | **Age**  **(SD)** | **LVEF**  **(%)** | **n**  **(M/F)** | **Age**  **(SD)** | **LVEF**  **(%)** |  |  |
| Hawkins 2018  USA | L4 PMI (below 25th gender specific percentile) | 240  (143/97) | 60  (36/24) | 81 (median – 77-85) | 57 (50-63)  (EF not LVEF) | 180  (107/73) | 80 (median- 75-85) | 57 (43-63)  (EF not LVEF) | 5.5 | CT |
| Okamura 2020  Japan | L3 PMI (below 25th sex-specific percentile) | 304  (253/51) | 76  (63/13) | 69.9 ± 8.9 | 55.5 ± 14.4 | 228  (190/38) | 66.6 ± 9.7 | 56.4 ± 13.9 | 5 | CT |
| Lopez  2019  USA | L3 PMI  Lowest tertile (<5.39 cm^2^ in males and <3.66 cm^2^ in females) | 160  (111/49) | 52  (36/16) | 72.8 ± 11 | <40 | 108  (75/33) | 63.2 ± 13.9 | <40 | 1 | CT |
| Yoon  2020  Korea | L3 PMI  Lowest tertile  (≤38.9 cm^2^/m^2^ in males and ≤31.3 cm^2^/m^2^ in females) | 522  (258/264) | 174  (86/88) | 80.6 ± 5 | 58.3 ± 11.1 (EF not LVEF) | 174  (86/88) | 77.7 ± 5.2 | 58.9 ± 10.5  (EF not LVEF) | 1 | CT |
| Saji  2016  Japan | L4 PMI  Lowest tertile (Males: tertile 1, 1,708-1,178 mm^2^/m^2^; tertile 2, 1,176-1,011 mm^2^/m^2^; tertile 3, 1,009-587 mm^2^/m^2^) (Females: tertile 1, 1,436-962 mm^2^/m^2^; tertile 2, 952-807 mm^2^/m^2^; tertile 3, 806-527 mm^2^/m^2^) | 232  (132/100) | 77  (44/33) | 79.8 ± 9.4 | 45.9 ± 15.7 (EF not LVEF) | 77  (44/33) | 78.7 ± 9.2 | 50.6 ± 13.1  (EF not LVEF) | 6 months | CT |
| Wittmann  2021  Austria | L3 PMI  (≤856 mm^2^/m^2^ in males and ≤635 mm^2^/m^2^ in females) | 106  (96/10) | - | - | - | - | - | - | 30 days | CT |

ALM, appendicular skeletal muscle; BMI, body mass index; CT, computed tomography; F, females; HFmrEF; heart failure with mid-range ejection fraction; LVEF, left ventricular ejection fraction; M, males; PMI, psoas muscle index; SD, standard deviation.

Data are expressed as mean ± SD.

Data are expressed as median (IQR).
