# Supplementary material for: Are sarcopenia and its individual components linked to all-cause mortality in heart failure? A systematic review and meta-analysis
Source: Clin Res Cardiol. 2023 Dec 12;114(5):532–40. doi: 10.1007/s00392-023-02360-8 (PMC12058882; doi:10.1007/s00392-023-02360-8)
Supplement: Supplementary file 15 — Supplementary file15 (DOCX 18 kb) [file 392_2023_2360_MOESM15_ESM.docx]

**Table S5.** Study and participant characteristics of the included studies in the systematic review and meta-analysis examining the effect of slow gait speed on all-cause mortality.

| **Study**  **Year**  **Country** | **Sarcopenia or muscle dysfunction definition** | **Total *n*  (M/F)** | **Patients with sarcopenia or muscle dysfunction definition** | | | **Patients without sarcopenia or muscle dysfunction definition** | | | **Median**  **Follow-up**  **(years)** |
| --- | --- | --- | --- | --- | --- | --- | --- | --- | --- |
|  |  |  | ***n* (M/F)** | **Age**  **(SD)** | **LVEF**  **(%)** | **n**  **(M/F)** | **Age**  **(SD)** | **LVEF**  **(%)** |  |
| Lo  2015  USA | Gait Speed (<0.8 m/s) | 1119  (550/569) | 566  (329/224) | 76 ± 6 | - | 553  (221/345) | 73 ± 5 | - | 1 |
| Ozawa (Kitasato)  2021  Japan | Gait Speed (SGS ratio <0.527 SGS ratio) | 1247  (724/523) | 213  (109/104 | 78 (74-84) | 46 ± 17 | 1034  (615/419) | 75 (71-81) | 47 ± 17 | 1 |
| Konishi  2021b  Japan | Gait Speed (<0.8 m/s) | 942  (550/392) | 187  (132/55) | 80-86 | HFrEF: 30 ± 7  HFpEF: 61 ±10 | 755  (418/337) | 77-80 | HFrEF: 32 ± 8  HFpEF: 59 ± 9 | 1 |
| Matsuzawa 2013  Japan | Gait Speed (per 0.1 m/s gait speed increase) | 472  (388/84) | 158  (130/28) | 69.9 ± 10.8 | 50.3 ± 13 | 155  (127/28) | 58.5 ± 10.4 | 56.4 ± 9.8 | 5.5 |
| Tanaka  (Kitasato)  2019  Japan | Gait Speed (per 0.1 m/s gait speed increase) | 388  (228/160) | 194  (87/107) | 76.5 ± 8.4 | 41.6 ± 17.4 | 194  (141/53) | 73.1 ± 6.7 | 43.3 ± 16.1 | 2.1 |
| Rodriguez-Pascual  2017  Spain | Gait Speed (slowest 20% of the population was defined at baseline, based on time to walk 15 feet, adjusting for gender and standing height) | 497  (194/303) | - | - | - | - | - | - | 1 |
| Vidán  2016  Spain | Gait Speed (slowest 20% of the population was defined at baseline, based on time to walk 15 feet, adjusting for gender and standing height) | 416  (210/206) | - | - | - | - | - | - | 1 |
| Zheng  2021  China | Gait Speed (slowest 20% of the population was defined at baseline, based on time to walk 15 feet, adjusting for gender and standing height) | 443  (225/218) | - | - | - | - | - | - | 6 months |
| Ashikawa  2022  Japan | Gait Speed  (<0.98 m/s) | 489  (354/135) | - | - | - | - | - | - | 2 |
| Joseph  2017  USA | Gait Speed (walking 5m in >6 seconds) | 75  (56/19) | - | - | - | - | - | - | 3 |
| Kano  2017  Japan | Gait Speed (Slow: 0.5-0.83; Slowest <0.5 m/s) | 1256  (361/895) | - | - | - | - | - | - | 326 days |
| Martin-Sánchez 2017  Spain | Gait Speed (slowest 20% of the population was defined at baseline, based on time to walk 15 feet, adjusting for gender, and standing height) | 465  (182/283) | - | - | - | - | - | - | 30 days |
| Chiaranda  2013  Italy | Gait Speed  (per quartiles) Mean Quartiles: 0.94 (0.08) 1.13 (0.02)  1.27 (0.05) 1.53 (0.14) | 1255  (1255/0) | 316  (316/0) | 65 ± 9 | 53 ± 11 | 326  (326/0) | 57 ± 9 | 58 ± 10 | 8.2 years |
| Sanchis  2020  Spain | Gait Speed (slowest 20% of the population was defined at baseline, based on time to walk 15 feet, adjusting for gender and standing height) | 342  (196/146) | - | - | - | - | - | - | 8.7 |

F, females; HFmrEF; heart failure with mid-range ejection fraction; HFpEF, heart failure with preserved ejection fraction; HFrEF, heart failure with reduced ejection fraction; LVEF, left ventricular ejection fraction; M, males; PMI, psoas muscle index; SD, standard deviation; SGS, slow gait speed.

Data are expressed as mean ± SD.

Data are expressed as median (IQR).
