# Supplementary material for: Are sarcopenia and its individual components linked to all-cause mortality in heart failure? A systematic review and meta-analysis
Source: Clin Res Cardiol. 2023 Dec 12;114(5):532–40. doi: 10.1007/s00392-023-02360-8 (PMC12058882; doi:10.1007/s00392-023-02360-8)
Supplement: Supplementary file 16 — Supplementary file16 (DOCX 14 kb) [file 392_2023_2360_MOESM16_ESM.docx]

**Table S6.** Study and participant characteristics of the included studies in the systematic review and meta-analysis examining the effect of low SPPB on all-cause mortality.

| **Study**  **Year**  **Country** | **Sarcopenia or muscle dysfunction definition** | **Total *n*  (M/F)** | **Patients with sarcopenia or muscle dysfunction definition** | | | **Patients without sarcopenia or muscle dysfunction definition** | | | **Median**  **Follow-up**  **(years)** |
| --- | --- | --- | --- | --- | --- | --- | --- | --- | --- |
|  |  |  | ***n* (M/F)** | **Age**  **(SD)** | **LVEF**  **(%)** | **n**  **(M/F)** | **Age**  **(SD)** | **LVEF**  **(%)** |  |
| Chiarantini  2010  Italy | SPPB scores  (0, 1-4, 5-8) | 157  (79/78) | 0: 47  1-4: 33  5-8: 45 | - | - | 32 | - | - | 2.5 |
| Iwatsu  2022 | SPPB  (High vs Low based on Youden index (sensitivity + specificity - 1)) (≤10 in HFrEF, ≤ 9 in HFmrEF, and ≤ 8 in the HFpEF group) | 542  (305/237) | - | - | - | - | - | - | 2 |

F, females; HFmrEF; heart failure with mid-range ejection fraction; HFpEF, heart failure with preserved ejection fraction; HFrEF, heart failure with reduced ejection fraction; LVEF, left ventricular ejection fraction; M, males; SD, standard deviation; SPPB, short physical performance battery.

Data are expressed as mean ± SD.

Data are expressed as median (IQR).
