# Supplementary material for: Are sarcopenia and its individual components linked to all-cause mortality in heart failure? A systematic review and meta-analysis
Source: Clin Res Cardiol. 2023 Dec 12;114(5):532–40. doi: 10.1007/s00392-023-02360-8 (PMC12058882; doi:10.1007/s00392-023-02360-8)
Supplement: Supplementary file 17 — Supplementary file17 (DOCX 26 kb) [file 392_2023_2360_MOESM17_ESM.docx]

**Table S7.** Quality assessment of the included studies based on the Methodological Index for Non-Randomized Studies (MINORS) tool.

|  |  |  |  |  |  |  |  |  |  |  |
| --- | --- | --- | --- | --- | --- | --- | --- | --- | --- | --- |
| Author, Year | **Aim** | **Inclusion of consecutive patients** | **Prospective collection of data** | **Endpoints appropriate to the aim of the study** | **Unbiased assessment of the study endpoint** | **Follow-up period appropriate to the aim of the study** | **Loss to follow up less than 5%** | **Prospective calculation of the study size** | **Total** | **Risk of bias** |
| Ashikawa, 2022 | 2 | 2 | 1 | 2 | 2 | 2 | 0 | 0 | 11/16 | Low |
| Iwatsu,  2022 | 2 | 2 | 1 | 2 | 2 | 2 | 2 | 0 | 13/16 | Low |
| Katano,  2022 | 1 | 1 | 0 | 2 | 2 | 2 | 2 | 0 | 10/16 | Low |
| Saito,  2022 | 2 | 2 | 1 | 2 | 2 | 2 | 1 | 1 | 13/16 | Low |
| Sze,  2022 | 2 | 2 | 0 | 2 | 2 | 2 | 0 | 0 | 10/16 | Low |
| Eschalier, 2021 | 1 | 0 | 1 | 2 | 2 | 2 | 1 | 2 | 11/16 | Low |
| Konishi,  2021a | 1 | 2 | 1 | 2 | 2 | 2 | 0 | 0 | 10/16 | Low |
| Konishi.  2021b | 1 | 2 | 1 | 2 | 2 | 2 | 2 | 0 | 12/16 | Low |
| Ozawa,  2021 | 2 | 2 | 0 | 2 | 2 | 2 | 1 | 0 | 11/16 | Low |
| Parahiba,  2021 | 2 | 2 | 1 | 2 | 2 | 0 | 0 | 2 | 11/16 | Low |
| Wittman, 2021 | 2 | 2 | 1 | 2 | 2 | 0 | 0 | 0 | 9/16 | Some concerns |
| Zheng,  2021 | 2 | 2 | 0 | 1 | 2 | 2 | 0 | 0 | 9/16 | Some concerns |
| Castillo-Martinez,  2020 | 1 | 2 | 0 | 1 | 2 | 2 | 2 | 0 | 11/16 | Low |
| Okamura,  2020 | 1 | 2 | 1 | 2 | 2 | 2 | 2 | 0 | 12/16 | Low |
| Sanchis,  2020 | 1 | 2 | 0 | 2 | 2 | 2 | 2 | 2 | 13/16 | Low |
| von Haehling,  2020 | 2 | 2 | 1 | 2 | 2 | 2 | 2 | 0 | 13/16 | Low |
| Yoon,  2020 | 1 | 2 | 0 | 2 | 2 | 2 | 2 | 0 | 11/16 | Low |
| Lopez,  2019 | 2 | 2 | 1 | 2 | 2 | 2 | 0 | 0 | 11/16 | Low |
| Tanaka,  2019 | 2 | 2 | 2 | 2 | 2 | 2 | 0 | 0 | 12/16 | Low |
| Hawkins,  2018 | 2 | 1 | 0 | 1 | 2 | 2 | 0 | 0 | 8/16 | Some concerns |
| Tanaka,  2018 | 2 | 2 | 0 | 2 | 2 | 2 | 2 | 0 | 14/16 | Low |
| Joseph,  2017 | 1 | 2 | 1 | 1 | 2 | 2 | 2 | 0 | 11/16 | Low |
| Kano,  2017 | 2 | 2 | 0 | 1 | 2 | 2 | 0 | 0 | 9/16 | Some concerns |
| Martin-Sanchez,  2017 | 2 | 2 | 0 | 1 | 2 | 2 | 0 | 0 | 9/16 | Some concerns |
| Rodriguez Pascual,  2017 | 2 | 1 | 1 | 1 | 2 | 2 | 2 | 0 | 11/16 | Low |
| Saji,  2016 | 2 | 2 | 1 | 2 | 2 | 2 | 2 | 0 | 13/16 | Low |
| Vidan,  2016 | 2 | 2 | 0 | 2 | 2 | 2 | 2 | 2 | 14/16 | Low |
| Lo,  2015 | 1 | 2 | 2 | 2 | 2 | 2 | 2 | 0 | 13/16 | Low |
| Chiaranda,  2013 | 2 | 2 | 1 | 1 | 2 | 2 | 0 | 0 | 10/16 | Low |
| Matsuzawa,  2013 | 1 | 2 | 1 | 2 | 2 | 2 | 0 | 0 | 10/16 | Low |
| Chiarantini,  2010 | 2 | 2 | 1 | 2 | 2 | 2 | 2 | 0 | 13/16 | Low |
| Izawa,  2009 | 2 | 2 | 1 | 2 | 2 | 2 | 0 | 0 | 11/16 | Low |
